# Review of disaster management training: A case study of a South African university

**DOI:** 10.4102/jamba.v15i1.1342

**Published:** 2023-07-21

**Authors:** Olivia Kunguma, Tendai Mapingure

**Affiliations:** 1 Disaster Management Training and Education Centre for Africa, Faculty of Natural and Agricultural Sciences, University of the Free State, Bloemfontein, South Africa; 2 Northern Cape Department of Education, Vuyolwethu High School, Kimberley, South Africa

**Keywords:** disaster studies, Short Learning Programmes, skills development, knowledge transfer, disaster legislation, community of practice

## Abstract

The goal of this article was to review a short learning programme offered to disaster management graduates and practitioners intending to enhance their knowledge and skills in disaster management. Short Learning Programmes (SLPs) are a form of professional development offered to adults working to better their knowledge or skills in a particular area of expertise. Short Learning Programmes provide theoretical and practical skills that one can learn in a full-time course over a shorter time. Disaster studies have been fairly new in South Africa since the promulgation of the *Disaster Management Act*, 57 of 2002. Therefore, few institutions offer disaster studies, particularly SLPs in disaster management. The authors sought to qualitatively, critically and systematically review the content and activities of an SLP offered by the University of the Free State-Disaster Management Training and Education Centre for Africa (UFS-DIMTEC). The structure, content and activities are documented in this article.

**Contribution:**

The study contributes to disaster studies andragogy by reviewing a short learning programme training. The review aided in improving the current course and encouraged the development of similar training by other institutions as a disaster legislation implementation activity and growth of the academic disaster risk field.

## Introduction

The past decades have entertained a series of conferences that focused on disaster risk reduction (DRR) issues with the goal of global sustainable development. At the end of each conference, some government leaders would pen their signatures on international agreements such as the 1994 Yokohama Strategy for a Safer World, the 2005 Kobe Conference, the 2005–2015 Hyogo Framework for Action, and the 2015–2030 Sendai Framework for DRR. The international DRR agreements, developments and reforms also contributed to the enactment of South Africa’s *Disaster Management Act*, 57 of 2002 (DMA), followed by the National Disaster Management Framework of 2005 (NDMF). A plethora of DRR agreements, policies, and legislation meant a growing need for skilled human resources to implement them (Holloway 2009; Kunguma 2020). Conclusively, all DRR international agreements and South African disaster management statutes strongly encourage disaster management education, training and research (Republic of South Africa 2005). Because of this, several higher education institutions globally and in South Africa embarked on a journey to develop disaster studies curricula. However, the development and establishment of disaster training institutions have not been as substantial or booming compared with disaster occurrences. Several authors, policies and legislation have pointed out the need for DRR education. The existence of DRR education would evoke disaster risk knowledge, practice, actions and behaviours of change in communities (Apronti et al. 2015; Nakano et al. 2020; Republic of South Africa 2002, 2005; United Nations Educational, Scientific and Cultural Organization [UNESCO] 2011).

Like the DRR international agreements, the DMA and NDMF reverberate the significance of education, training and research. They promote DRR education in primary, secondary and tertiary education and community capacity building. Already, South Africa has progressed in developing graduate programmes in disaster management. Some of the institutions are the Disaster Management Training and Education Centre for Africa at the University of the Free State, the Disaster Mitigation for Sustainable Livelihoods Programme at the University of Cape Town, The Research Alliance for Disaster Risk Reduction (RADAR) at Stellenbosch University and the African Centre for Disaster Studies at the North-West University. The importance of DRR education is observed in the NDMF, identifying it as a supporting enabler, ‘Enabler 2 – Education, Training, Public Awareness and Research’ (Republic of South Africa 2002, 2005).

Furthermore, the South African National Disaster Management Centre (NDMC) conducted studies to determine practitioners’ education, training, research needs, and the establishment of DRR institutional arrangements. In 2008, a handbook on establishing foundational institutional arrangements for disaster risk management highlighted the need for a Portfolio Manager to handle education training, knowledge management, public awareness and research as key components in a disaster management centre. The handbook also states that training, media and public information centre should be a minimum requirement infrastructure of a disaster management centre to promote capacity building of volunteers and other stakeholders (Reid & NDMC 2008). In 2010, the NDMC did a National Education, Training and Research Needs and Resources Analysis (NETaRNRA) (NDMC 2010). The NETaRNRA is a foundation for developing an appropriate disaster management training and education programme responsive to changing risk conditions (Republic of South Africa 2005).

Furthermore, in 2022, the NDMC commenced a new programme termed ‘National Research Agenda’. It brings together all the higher education institutions and other stakeholders offering DRR education to ensure that DRR education and research are conducted uniformly (Department of Cooperative Governance Republic of South Africa 2022). What is currently lacking is a review of the DRR pieces of training to determine if they contribute to the DRR resilience of communities as much as they develop learners’ knowledge. Of particular focus in this study are Short Learning Programmes (SLPs), a form of professional development offered to already working adults to update or upgrade their knowledge or skills in a particular area of expertise.

For such pieces of training to make DRR and resilience contributions to communities, they must be based on an underlying theoretical basis. Therefore, participation in a community of practice approach is of interest and applicable to the issue under investigation. This approach has been identified by Nakano et al. (2020) as grounded in the theory of legitimate peripheral participation (LPP) postulated by Lave and Wenger (1991). In this LPP approach, the significant aspect of learning is the interrelationships of roles between the mentor and the learners. The approach is a learning contextual social process achieved through participation in a community. The participants join a community in the periphery and learn through observation. In the end, the participants become experienced members of the community practices. The DRR is envisaged to stimulate proactive attitudes in learners through active participation, where learning is perceived as a transformation of identity through the involvement of the learners in practices developed by the instructor (Nakano et al. 2020). Participation in a community of practice andragogy has been implemented in the DRR SLPs offered by various institutions, enabling both the instructors and learners to develop their personalities and comprehension through active participation and engagements with others as they interact and create relationships. Nakano et al. (2020), Willms (2003), and Osterman (2000) suggest that the legitimate participation in the communities instils a sense of belonging in the learners’ minds as they actively engage, imagining themselves, their roles, and the future they envisage in the communities. Therefore, the participants’ skills are sharpened with practical experience in communities where DRR activities will be implemented; thus, knowledge will be aligned with practice. Furthermore, the community of practice involves volunteers who participate and engage in shared concerns such as practical experiences, developing and discussing new areas of interest, and building a sense of community (Floding & Swier 2012).

With all of the aforementioned, this study reviews the SLP offered by the University of the Free State – Disaster Management Training and Education Centre for Africa (UFS-DiMTEC) in collaboration with the United Nations University – Institute for Environment and Human Security (UNU-EHS). The course is titled ‘Block Course-From Vulnerability to Resilience’. Every year there is a new theme to fit the disaster trend. Table 1 illustrates several themes focused on in the past years.

**TABLE 1:** Themes of some block courses.

Number	Theme	Year
1	‘Reducing vulnerability and strengthening Coping and Adaptation Strategies for Sustainable Development’	2018
2	‘Managing the complexities of dynamic human communities: Towards 2030 global agenda for Sustainable Livelihood Development’	2019
3	‘Disaster Risk Management for Sustainable Development’	2020
4	COURSE NOT HELD DUE TO COVID-19 RESTRICTIONS	2021
5	‘Sustainable resilience building as we emerge from the complex global pandemic: Towards 2030 global agenda for Sustainable Livelihood Development’	2022

The SLP or Block Course is held annually with a strong focus on building resilience, sustainable development, and the reduction of vulnerabilities. The SLP enrols different learners or participants from various work or academic disciplines and sectors. Through engagement in natural community settings (through the field exercise), the participants allow for knowledge sharing and skills development, thus promoting innovative ideas in a real-life situation in a practical manner. The community of practice approach in DRR training fosters long-term evaluation of knowledge (Gill et al. 2019).

Firstly, this article aims to appraise and critically review the SLP to determine if its contents and activities contribute to DRR resilience building in communities. Secondly, to provide practical recommendations that aid in the betterment and development of disaster management SLPs.

## Methodology and description of the course

The study applied a literature review methodology of journal articles to conceptualise the article better. The authors informally observed (course observation) (Qaddo 2019) the course over 2 weeks to loosely document the course’s contents, structure, and activities thematically (Boudah 2011). Volunteers from the course participants were asked to share their perceptions of the course. One facilitator and two participants agreed to share their perceptions. The study aimed to appraise the SLP’s content, andragogy and activities as a potential good practice. It did not focus on the findings of the participants in the fieldwork.

### About the Short Learning Programme

The course offered by UFS-DiMTEC and UNU-EHS is a strategic collaboration that offers participants a local and international perspective on DRR. The course was held at the University of the Free State, Bloemfontein Campus, in Mangaung Metropolitan Municipality (MMM), Bloemfontein City, Free State Province of South Africa. It was over 2 weeks and included theory (knowledge transmission approach) and a practical exercise. The course coordinators approached the local disaster management centre (MMM Disaster Management Centre) to assist with the practical exercise. The MMM Disaster Management Centre agreed to be part of the SLP. Their role entailed identifying a vulnerable community and making appointments with the community leaders to inform them of the SLPs participants’ visit to the community. The course coordinator’s role also included escorting the SLP participants to the community and attending the participants’ practical exercise presentations after the field visit.

Mangaung Metropolitan Municipality is the local municipality that governs Bloemfontein, Thaba Nchu, Botsabelo and other towns within the Free State Province of South Africa. The vulnerable community that MMM identified was Khayelitsha informal settlement. It is an informal settlement within Bloemfontein. Khayelitsha, established in 2005, which comprises informal housing with 296 people and formal housing with 3589 people. The study focused on the informal residence (informal settlement) in the periphery of Khayelitsha. The informal housing area has limited access to clean drinking water, no electricity, no waste management, and pit toilets for sanitation. Most of the population is unemployed and those employed work in the informal sector (Mangaung Metropolitan Municipality 2022).

After the course was advertised, 17 participants attended the SLP. The participants were from Namibia, Zimbabwe, Uganda, South Africa, South Sudan, and Cameroon. The participants were PhD candidates, Masters in disaster management students, and specialists working for private institutions, non-governmental organisations, and disaster management centres.

## Results and discussion

The results are based on the course observation and engagement with volunteer facilitators and participants. In addition, the use of COVID-19 as a case study was imperative because the use of real and current trends. In addition, the use of COVID-19 as a case study was imperative because the use of real and current trends provides a concrete application to knowledge and skills in learning and the learners can also relate (InteGrate 2023).

### Content and activities of the course

The content of the course included the following topics but was not limited to:

- Disaster management terminology.
- Ecosystems in DRR.
- Frameworks in disaster management.
- Disaster risk assessment.
- International disaster management legislation.

The various facilitators from the University of the Free State, DRR Consultants, United Nations University (Bonn, Germany) and National Public University (Hungary) trained the participants on various research designs and methodologies, including how to engage with communities. In the last week of the course, the facilitators guided the participants in developing a data collection tool. The researchers qualitatively observed how the participants transitioned from the classroom theory to developing an interview guide for data collection. The participants engaged 15 randomly selected community members on coronavirus disease 2019 (COVID-19) socio-economic issues. The engagement was aligned with the SLP theme, as stated earlier. Training comes to life through fieldwork; the participants can connect with the community context and turn theory into practice (The South African College of Applied Psychology [SACAP] 2019; Tippa & Mane 2019). The participants analysed the data and presented it to a local disaster management centre, intending for them to incorporate the information into their COVID-19 risk reduction, response and recovery planning. In their qualitative study on enhancing learning by integrating theory and practice, Wrenn and Wrenn (2009) support the practice of a learner serving as a teacher. This practice was observed when the course participants engaged the local centre.

The participants decided to use COVID-19 as a case study because 2 years into the pandemic, various stakeholders have been convening to solicit strategies on how best to start living with COVID-19 and rebuild what the virus destroyed. As COVID-19 was declared a global pandemic (WHO 2020), governments have been proactive in organising conventions and institutions to discuss COVID-19 risk reduction strategies and strategies to start living with the virus. Regrettably, most of the conventions are policy-related with limited engagement with the community. They focused on developing and reviewing policies for responding to COVID-19 (Organisation for Economic Co-operation and Development [OECD] 2022). Few government interventions focused on acquiring COVID-19 socio-economic perceptions from the affected communities; most of the interventions were through individual researchers (Ashraf 2020; Nicola et al. 2020). During the observation of participants engaging in discussions, the participants argued that the government did not engage with societies meaningfully and hold sustainable dialogues with multiple stakeholders. The participants, therefore, decided to explore the space of engaging the societies.

The authors asked for volunteers from the 17 participants to provide their perceptions of the course. Two participants and one facilitator agreed to share their perceptions with the following verbatim comments:

‘The multi-disciplinary nature of the course in terms of the content covered provided the participants with diverse theoretical and practical information to contribute to the betterment of their areas of study, work disciplines and living environments. Also, the interactive nature and participatory approaches are some of the uniqueness of this course that indulges students and prepares them to further their studies and enhance their research skills. The local disaster centre’s educational tour gave them first-hand information on disaster-managed operations. The fieldwork consisting of the Rapid Risk Analysis in a selected informal settlement gave the students the practical aspects entailed in the empirical research process for applications in their studies. In addition, with the different cultural background of the participants in the community they were involved, adjustments, cooperation and teamwork was built as well as understanding and developing human relations skills; at the same time, understanding and appreciating different cultural backgrounds of communities was fostered. I believe the participants returned to their portfolios better equipped with theoretical and practical disaster managing skills,’ explained one of the Facilitators.’ (Participant 1, Facilitator, 07/03/2022)

The facilitator’s comment indicates that the course aims to build community resilience through the participants’ areas of study, work, and communities. In their book, ‘Contributions to the local community through the University’, Maeshima (2022) argues that experts with different skills from universities should return the knowledge they acquired through education to communities as a social contribution, which builds community resilience to risks. Another comment was shared by a participant as follows:

‘COVID-19 taught us that we need to build resilience to survive. Also, the topics covered in the course appealed to me. They were relevant to my PhD study on investing in resilience and DRR to reduce food insecurity among smallholder farmers in South Africa. The knowledge I gained from the course will help me achieve my PhD goals, especially using resilience frameworks. My study aims to equip smallholder farmers with knowledge and bridge the gap between DRR, Resilience and food insecurity.’ (Participant 2, Learner, 07/03/2022)

Concerning the field visit, one of the participants said:

‘Khayelitsha is a great community. I saw the potential for a stable and secure community, but the people will need government support. They are industrious and have learnt to be resilient and cope with hazards independently. They are receptive and open people who are ready for help. I hope the government will consider this community and do something tangible for them. No single sector will be able to respond alone to the depth and breadth of humanitarian crises. Progress will need strong support from state and civil society at large.’ (Participant 3, Learner, 07/03/2022)

### Application of participation in a community of practice approach

To engender continuous DRR education in SLPs, participation in a community of practice approach, a proactive attitude paradigm needs to be explored because proactive attitudes in participants are elicited. Participant 2’s comment shows that through community engagement, they could identify the community’s needs and recommend possible solutions. Proactiveness includes anticipating problems and needs and investing in plans that prevent or mitigate disasters’ impact (Committee on Science, Engineering, and Public Policy 2012). Through the SLP, the participants gained theoretical knowledge about DRR. This was assessed through the report they presented to the local municipality and the facilitators, including the study area’s empirical findings. Subsequently, the participants engaged with the local disaster management centre and community members about the risks they are exposed to and their expectations from the government. The fieldwork engagement was informed by the theoretical knowledge gained from the classroom. By engaging with disaster officials and communities at risk, the participants can gain knowledge of their experiences in DRR.

The SLP content and activities support proactive attitudes, as illustrated in participating in a community of practice approach.

## Conclusion

The course allowed the participants to provide the community of Khayelitsha with a voice and connect them with the policymakers. The participants presented their field visit results and shared them with the Mangaung Metropolitan Municipality Disaster Management Centre. This approach was with the intention that the information would better equip the policymakers with custom-made decisions to assist the community with risk reduction measures better. The practical exercise was more of a pilot study from which the MMM officials could take over and conduct a full study. The findings highlight the significance of investing in knowledge sharing to promote effective and acceptable public-informed policies. When such policies are implemented, they yield sustainable resilience to future COVID-19 breakouts or new pandemic-related disasters. There is a need for the government to broaden the science base and community participation when making policies.

Although not evaluated, based on the course activities, the article demonstrates that this andragogical approach, which is theoretical and practical, is beneficial to participants, policymakers, and at-risk communities. The philosophy of incorporating community involvement in the SLP influences student learning, bridging the gap between theory and practice, thereby giving higher education institutions the desired outcome. The course coordinators strive to achieve the principles of good practice to provide the best learning experience to the participants through field-based experiences in communities that are different from their usual ones. By adopting the theory of practice approach in disaster management training, the role players in DRR, namely the researchers, communities, universities and policymakers, will develop their knowledge and practical experience. This exposure of participants to real-life situations empowers them with the competence and skills to identify their strengths and weaknesses. It also allows them to work on their weaknesses and enrich their strengths in their specialised field. Such experiences serve as the basis for the course coordinators and the university to assess the quality of training offered to learners.
